# Supplementary material for: Efficacy and Effect on Liver Functional Reserve of Atezolizumab and Bevacizumab for Unresectable Hepatocellular Carcinoma in Patients Who Do Not Meet Eligibility Criteria of IMbrave150
Source: Cancers (Basel). 2022 Aug 15;14(16):3938. doi: 10.3390/cancers14163938 (PMC9405784; doi:10.3390/cancers14163938)
Supplement: Supplementary file 1 [file cancers-14-03938-s001.zip › cancers-1829074-supplementary.pdf]

Supplementary Table S1

	IMbrave150 in (n=43)	IMbrave150 out (n=72)
Interruption of Atezo n, (%)	1 (2.3)	16 (22.2)
Reason of Interruption (Atezo)		
HCC rupture n, (%)	0 (0.0)	2 (2.8)
Liver dysfunction n, (%)	0 (0.0)	2 (2.8)
Fever up n, (%)	0 (0.0)	2 (2.8)
Diarrhea n, (%)	0 (0.0)	1 (1.4)
General fatigue n, (%)	0 (0.0)	1 (1.4)
Pneumonia n, (%)	0 (0.0)	1 (1.4)
Ascites n, (%)	0 (0.0)	1 (1.4)
Acute renal failure n, (%)	0 (0.0)	1 (1.4)
Appetite loss n, (%)	1 (2.3)	0 (0.0)
Unknown n, (%)	0 (0.0)	2 (2.8)
Others n, (%)	0 (0.0)	5 (6.9)
Interruption of Bev n, (%)	8 (18.6)	22 (30.6)
Reason of Interruption (Bev)		
Proteinuria n, (%)	5 (11.6)	5 (6.9)
Pneumonia n, (%)	0 (0.0)	2 (2.8)
Fever up n, (%)	0 (0.0)	2 (2.8)
Liver dysfunction n, (%)	0 (0.0)	2 (2.8)
General fatigue n, (%)	0 (0.0)	1 (1.4)
Thrombocytopenia n, (%)	0 (0.0)	1 (1.4)
Diarrhea n, (%)	0 (0.0)	1 (1.4)

Hypertension n, (%)	0 (0.0)	1 (1.4)
Appetite loss n, (%)	1 (2.3)	0 (0.0)
Ascites n, (%)	0 (0.0)	1 (1.4)
Acute renal failure n, (%)	0 (0.0)	1 (1.4)
Unknown n, (%)	1 (2.3)	2 (2.8)
Others n, (%)	1 (2.3)	4 (5.6)

	**Child-Pugh A (n=106)**	**Child-Pugh B (n=9)**
Interruption of Atezo n, (%)	13 (12.3)	4 (44.4)
Reason of Interruption (Atezo)		
HCC rupture n, (%)	1 (0.9)	1 (11.1)
Liver dysfunction n, (%)	2 (1.9)	0 (0.0)
Fever up n, (%)	2 (1.9)	0 (0.0)
Diarrhea n, (%)	1 (0.9)	0 (0.0)
General fatigue n, (%)	0 (0.0)	1 (11.1)
Pneumonia n, (%)	1 (0.9)	0 (0.0)
Ascites n, (%)	1 (0.9)	0 (0.0)
Acute renal failure n, (%)	1 (0.9)	0 (0.0)
Appetite loss n, (%)	1 (0.9)	0 (0.0)
Unknown n, (%)	0 (0.0)	2 (22.2)
Others n, (%)	5 (4.7)	0 (0.0)
Interruption of Bev n, (%)	26 (24.5)	4 (44.4)
Reason of Interruption (Bev)		
Proteinuria n, (%)	10 (9.4)	0 (0.0)
Pneumonia n, (%)	2 (1.9)	0 (0.0)

Fever up n, (%)	2 (1.9)	0 (0.0)
Liver dysfunction n, (%)	2 (1.9)	0 (0.0)
General fatigue n, (%)	0 (0.0)	1 (11.1)
Thrombocytopenia n, (%)	0 (0.0)	1 (11.1)
Diarrhea n, (%)	1 (0.9)	0 (0.0)
Hypertension n, (%)	1 (0.9)	0 (0.0)
Appetite loss n, (%)	1 (0.9)	0 (0.0)
Ascites n, (%)	1 (0.9)	0 (0.0)
Acute renal failure n, (%)	1 (0.9)	0 (0.0)
Unknown n, (%)	1 (0.9)	2 (22.2)
Others n, (%)	5 (4.7)	0 (0.0)

	**Platelet ≧ 7.5×10^4 /μl (n=108)**	**Platelet < 7.5×10^4/μl (n=7)**
Interruption of Atezo n, (%)	14 (13.0)	3 (42.9)
Reason of Interruption (Atezo)		
HCC rupture n, (%)	2 (1.9)	0 (0.0)
Liver dysfunction n, (%)	2 (1.9)	0 (0.0)
Fever up n, (%)	2 (1.9)	0 (0.0)
Diarrhea n, (%)	1 (0.9)	0 (0.0)
General fatigue n, (%)	1 (0.9)	0 (0.0)
Pneumonia n, (%)	1 (0.9)	0 (0.0)
Ascites n, (%)	1 (0.9)	0 (0.0)
Acute renal failure n, (%)	1 (0.9)	0 (0.0)
Appetite loss n, (%)	0 (0.0)	1 (14.3)
Unknown n, (%)	1 (0.9)	1 (14.3)

Others n, (%)	4 (3.7)	1 (14.3)
Interruption of Bev n, (%)	26 (24.1)	4 (57.1)
Reason of Interruption (Bev)		
Proteinuria n, (%)	10 (9.3)	0 (0.0)
Pneumonia n, (%)	2 (1.9)	0 (0.0)
Fever up n, (%)	2 (1.9)	0 (0.0)
Liver dysfunction n, (%)	2 (1.9)	0 (0.0)
General fatigue n, (%)	1 (0.9)	0 (0.0)
Thrombocytopenia n, (%)	0 (0.0)	1 (14.3)
Diarrhea n, (%)	1 (0.9)	0 (0.0)
Hypertension n, (%)	1 (0.9)	0 (0.0)
Appetite loss n, (%)	1 (0.9)	0 (0.0)
Ascites n, (%)	0 (0.0)	1 (14.3)
Acute renal failure n, (%)	1 (0.9)	0 (0.0)
Unknown n, (%)	2 (1.9)	1 (14.3)
Others n, (%)	4 (3.7)	1 (14.3)

Atezo: Atezolizumab, Bev: bevacizumab

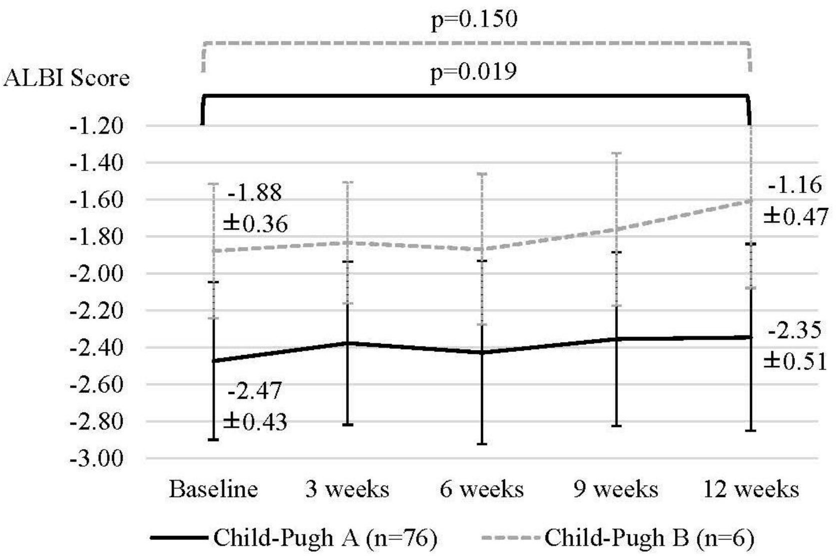

mALBI grade	Child-Pugh A	Child-Pugh B
Non-deteriorated, n	54	6
Deteriorated, n	22	0
p-value	p=0.185	

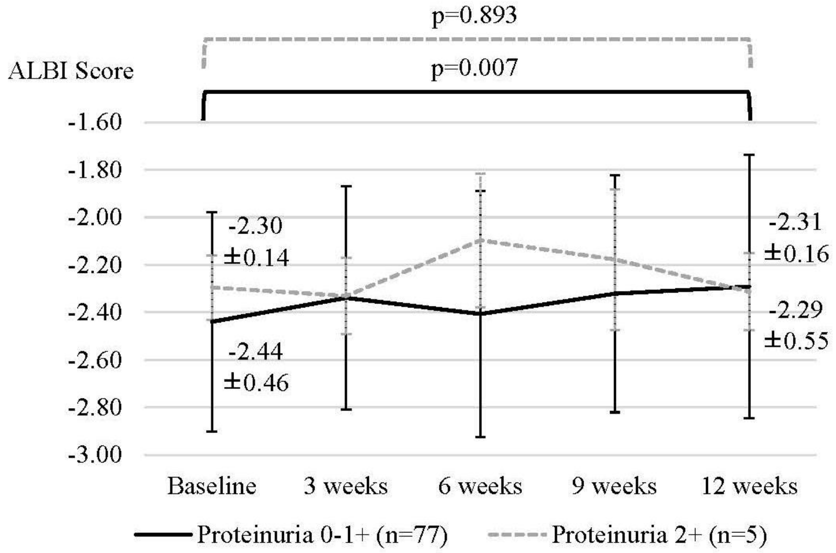

mALBI grade	Proteinuria 0-1+	Proteinuria 2+
Non-deteriorated, n	54	4
Deteriorated, n	19	1
p-value	p=1.000	

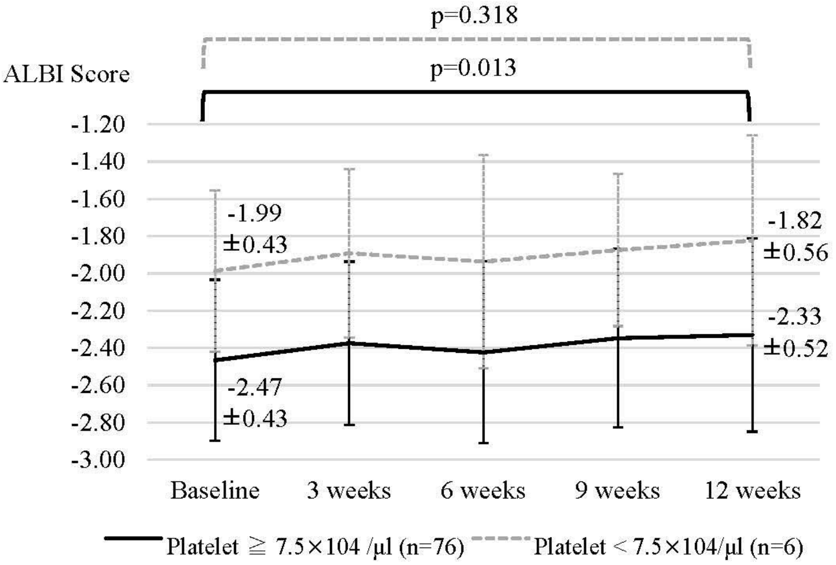

mALBI grade	Platelet ≧ 7.5 × 10^4 /μ	Platelet < 7.5 × 104/μl
Non-deteriorated, n	55	5
Deteriorated, n	21	1
p-value	p=1.000	

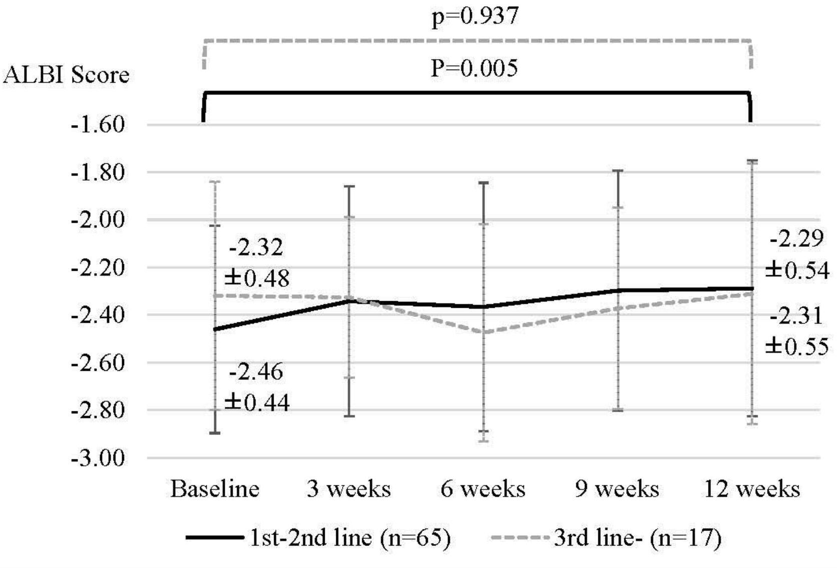

mALBI grade	1st -2nd line	3rd line-
Non-deteriorated, n	46	14
Deteriorated, n	19	3
p-value	p=0.539	

Figure S1. Changes in the ALBI score and mALBI grade during atezolizumab and bevacizumab treatment for unresectable HCC in each subgroup. mALBI: modified albumin-bilirubin grade, BCLC: Barcelona Clinic Liver Cancer, mALBI: modified albumin-bilirubin grade, Vp: portal vein invasion.
